# Personality is tightly coupled to vasopressin-oxytocin neuron activity in a gregarious finch

**DOI:** 10.3389/fnbeh.2014.00055

**Published:** 2014-02-25

**Authors:** Aubrey M. Kelly, James L. Goodson

**Affiliations:** Department of Biology, Indiana UniversityBloomington, IN, USA

**Keywords:** vasopressin, oxytocin, social behavior, anxiety, personality, vasotocin, mesotocin

## Abstract

Nonapeptides of the vasopressin-oxytocin family modulate social processes differentially in relation to sex, species, behavioral phenotype, and human personality. However, the mechanistic bases for these differences are not well understood, in part because multidimensional personality structures remain to be described for common laboratory animals. Based upon principal components (PC) analysis of extensive behavioral measures in social and nonsocial contexts, we now describe three complex dimensions of phenotype (“personality”) for the zebra finch, a species that exhibits a human-like social organization that is based upon biparental nuclear families embedded within larger social groups. These dimensions can be characterized as Social competence/dominance, Gregariousness, and Anxiety. We further demonstrate that the phasic Fos responses of nonapeptide neurons in the paraventricular nucleus of the hypothalamus and medial bed nucleus of the stria terminalis are significantly predicted by personality, sex, social context, and their interactions. Furthermore, the behavioral PCs are each associated with a distinct suite of neural PCs that incorporate both peptide cell numbers and their phasic Fos responses, indicating that personality is reflected in complex patterns of neuromodulation arising from multiple peptide cell groups. These findings provide novel insights into the mechanisms underlying sex- and phenotype-specific modulation of behavior, and should be broadly relevant, given that vasopressin-oxytocin systems are strongly conserved across vertebrates.

## Introduction

In humans, personality profoundly impacts the neural processing of social stimuli (Kennis et al., 2013), but to our knowledge, mechanisms of similarly complex behavioral phenotypes have not been examined at high resolution (i.e., at a cellular level) in nonhuman species. Here we provide the first extensive description of complex phenotype structure in the highly social zebra finch (*Taeniopygia guttata*), a popular species for genomic and behavioral studies, and explore the ways in which neural processing of social stimuli varies in relation to phenotype. Because the vasopressin-oxytocin (VP-OT) nonapeptides are well known for their modulation of social behavior and physiological stress response, and often exert sex- and phenotype-specific effects (Veenema and Neumann, 2008; Young, 2009; Goodson and Thompson, 2010), we hypothesized that the responses of nonapeptide neurons to social novelty vary in relation to sex and dimensions of personality.

The vertebrate VP-OT systems are strongly conserved, and homologous cell groups are recognized across all vertebrate groups. There are two clades of vertebrate nonapeptides that are evolutionarily derived from a duplication of the arginine vasotocin (VT) gene. One of these clades includes the mammalian forms of OT and the avian homolog Ile^8^-OT (mesotocin, MT), and the second includes the mammalian forms of VP and the nonmammalian homolog VT (Ile^3^-VP). Peptides in both lineages are important modulators of vertebrate social behavior (Young, 2009; Goodson and Thompson, 2010; Neumann et al., 2010; Albers, 2012).

Importantly, there is considerable variation in nonapeptide function and anatomy that is linked to differences in social behavior across sexes, species and individuals (Goodson, 2005). Nonapeptide receptor distributions vary in relation to mating system in voles, and in relation to grouping behavior in finches (Hammock et al., 2005; Goodson et al., 2006; Ophir et al., 2012). Finch group-size preferences are reflected in the activity of VT neurons in the medial bed nucleus of the stria terminalis (BSTm; a component of the medial extended amygdala), which are sensitive to social valence. These cells increase their Fos activity selectively in response to positive social stimuli, and thus, whereas same-sex stimuli increase the transcriptional activity of these VT neurons in gregarious species, the opposite is observed in territorial species (Goodson and Wang, 2006). In addition, nonapeptide anatomy and function vary in relation to sex across the vertebrate classes (Carter, 2007; De Vries, 2008; Goodson, 2008). Dimorphism in the VT/VP cell group of the BSTm is often extreme, with males having far more cells and denser projections than females (De Vries and Panzica, 2006). These neurons are highly sensitive to sex steroids in most species, but only modestly so in the opportunistically breeding zebra finch, which maintains a large number of cells year-round (De Vries et al., 1985; Kabelik et al., 2010). Nonapeptide functions, anatomy, and/or release also vary in relation to anxiety phenotype (Neumann et al., 2010), dominance-subordinance status (Caldwell et al., 2010; Mooney and Holmes, 2013), courtship phenotype (Goodson et al., 2009c), pair-bond status (Young and Wang, 2004), and developmental experiences related to parenting, social environment, and stress (Champagne et al., 2001; Curley et al., 2009; Veenema, 2012). To date, variation in the VT/VP circuitry arising in the BSTm has been most extensively linked to variation in affiliation behaviors, whereas VT/VP and OT neurons of the paraventricular nucleus of the hypothalamus (PVN) are more strongly linked to variation in developmental stress and anxiety (Wigger et al., 2004; Goodson and Thompson, 2010; Veenema, 2012; Zhang et al., 2012).

A growing body of work in humans likewise suggests that VP and OT play important roles in social cognition and behavior, although the lack of antagonist studies limits interpretations. Intranasal delivery of VP increases altruism and modulates neural processes related to social memory and recognition of emotion (Rilling et al., 2012; Zink and Meyer-Lindenberg, 2012), whereas intranasal OT enhances social and emotion recognition, and facilitates interpersonal relations, including cooperation and trust (Kosfeld et al., 2005; Zak et al., 2007; Guastella et al., 2008, 2010; Rimmele et al., 2009; Andari et al., 2010; Bartz et al., 2010b; Hurlemann et al., 2010). However, pre-existing attachment representations and individual differences in social proficiency moderate OT effects on empathy and social memory-related task performance, demonstrating that personality influences nonapeptide effects on behavior and cognition (Bartz et al., 2010a,b, 2011; Graustella and Macleod, 2012).

In the present experiments we first quantify the personality profiles of male and female zebra finches, and subsequently examine Fos protein expression (a proxy marker of neural activity) in VT and MT neurons following exposure to different social contexts.

## Materials and methods

### Animals

Forty male and forty female zebra finches were used for these experiments. Subjects were obtained as adults from a commercial supplier and housed in same-sex groups of 6–10 except for 3 days of testing in colonies, which contained four males and four females. Subjects were kept on a 14L:10D photoperiod with full spectrum lighting and were provided with finch seed mix, cuttlebone, grit, and water *ad libitum*. Tests were conducted in a humane manner and in full compliance with all federal and institutional regulations.

### Behavioral testing

All behavioral assays listed below were conducted in the same order for all subjects. With the exception of colony observations, which are more time consuming, each assay was completed within a 2-week period for all animals. Colony observations were completed over a 6-week period. Note that most of the assays are sufficiently short and simple (e.g., 6 min novel-familiar choice tests; or exposure to a novel cage) that they are likely no more salient than a regular cage change, and hence should not produce carry-over effects that impact subsequent measurements. The only assay that we expect to produce carry-over effects is the colony test, given that colony testing involves intense courtship, aggression, and other social behaviors over multiple days. We therefore conducted the colony tests last.

#### Novelty-suppressed feeding

Food was removed from subjects' cages prior to lights-on in the morning. After lights-on, subjects were placed in a novel cage (31 cm W × 20 cm H × 36 cm D) that contained a novel purple Nitrile glove hanging above a food dish. Subjects were video recorded for 30 min and the latencies to move and feed were quantified.

#### Exploration of a novel environment

Subjects were placed in a flight cage (1.3 m W × 1.8 m H × 1.8 m D) with a branch cluster in each of the four corners for 4 min. We recorded the latency to move and number of branch clusters explored.

#### Group size preference

Subjects were placed into a 1 m wide (0.43 m H × 0.36 m D) cage that was divided into seven zones by perches (Kelly et al., 2011). The perches at each end of the cage were approximately 4 cm from the cage wall, which adjoined a 0.5 m wide (0.43 m H × 0.36 m D) cage containing two novel same-sex stimulus birds at one end and 10 novel same-sex stimulus birds at the other (sides counterbalanced across subjects). Subject location was recorded every 15 s for 5 min. Time spent with the large and small groups was defined as time spent on the perch closest to the stimulus cage.

#### Novel vs. familiar social preference

Using the same cage arrangement as just described for tests of group size preference, subjects were exposed to cages containing five novel same-sex conspecifics and five familiar same-sex cagemates. Subject location was recorded every 15 s for 5 min, with sides counterbalanced across subjects. Time spent with the novel and familiar groups was defined as time spent on the perch closest to the stimulus cage.

#### Colony observations

Behavioral observations in colonies were conducted as previously described (Kabelik et al., 2009; Goodson et al., 2012b; Klatt and Goodson, 2013). Four subjects of each sex, all novel to each other, were moved into colony cages (1.3 m W × 0.43 m H × 0.36 m D), each containing four nest cups and shredded burlap nesting material. Focal observations were conducted 6 times over 3 days (AM/PM). Session 1 observations were 5 min per subject and began 10 min after the establishment of colonies, and Sessions 2–6 were 10 min each. The shorter observation period for Session 1 allows for the quantification of behavior in all subjects during the initial burst of courtship and competitive aggression. Social and nesting behaviors quantified were allopreen, follow, directed song, dances, copulation, undirected song, pick up nest item, carry nest item to nest, time spent on nest, and latency to pair bond. Aggressive behaviors quantified were displacements, threats, beak fences, and pecks, and we also quantified displacements received from other birds. As in previous studies, all data except pairing were converted to units of behavior per minute not spent on the nest (Kabelik et al., 2009; Goodson et al., 2012b; Klatt and Goodson, 2013).

### Principal components analysis

Given the large number of behaviors that were quantified, we chose to employ principal components analysis (PCA), which allows three or more variables to be reduced to a smaller number of factors that account for most of the variance observed in the full set of measured variables (Wuensch, 2012). Note that although variables that load onto a given principal component (PC) are statistically related, the relationship is not necessarily linear. PCA was initially conducted separately for males and females. A loading of ≤−0.4 or ≥0.4 is generally considered strong (Wuensch, 2012), and we therefore took the conservative approach of eliminating variables that did not load ≤−0.300 or ≥0.300 on any of the sex-specific principal components (PCs). This removed numerous low-frequency behaviors (e.g., copulation and allopreen). The first three PCs for both males and females exhibited strong eigenvalues ranging (1.868–3.832) and were very similar in structure (Supplemental Tables 1, 2). Hence, given the similar structures of the male and female PCAs, and our need to directly compare the sexes, we conducted a final PCA using data from all subjects, excluding male-specific behaviors (no female-specific behaviors were quantified). In our naming and interpretations of the final PCs, we conservatively focus on the variables that load most strongly (≤−0.5 or ≥0.5), which we have bolded in Table 1. The strength of the PC approach is demonstrated by the fact that the PCs generated in this analysis extensively and significantly predict VT- and MT-Fos colocalization.

**Table 1:** **Principal components matrix of zebra finch behavior^1^**.

	**PC1 “Social competence/dominance”**	**PC2 “Gregariousness”**	**PC3 “Anxiety”**
**BEHAVIORAL MEASURES**			
**Colony behaviors**			
Displacements, session 1	0.113	−0.330	0.384
Displacements, sessions 2–6	**0.769**	−0.235	0.062
Displaced by others, session 1	−0.379	−0.208	−0.256
Displaced by others, sessions 2–6	−**0.595**	−0.233	−0.199
Threats	**0.685**	−0.145	−0.204
Latency to pair bond	−**0.560**	0.255	0.289
Time on nest	**0.740**	−0.146	−0.263
**Choice tests**			
Time with novel birds	−**0.543**	−0.437	0.076
Time with familiar birds	**0.501**	0.168	0.053
Time with small group	0.001	−**0.769**	−0.091
Time with large group	0.094	**0.759**	0.003
**Anxiety tests**			
Latency to move (feed)	−0.052	0.290	0.461
Latency to feed	0.160	0.140	**0.706**
Latency to move (explore)	0.155	−0.244	**0.641**
Branches explored	−0.028	0.376	−**0.598**

1 PC structures were very similar in males and females, and sexes are shown combined. Loadings of ≥0.500 or ≤−0.500 are bolded.

### Behavioral manipulations for Fos analyses

**Figure 1:**
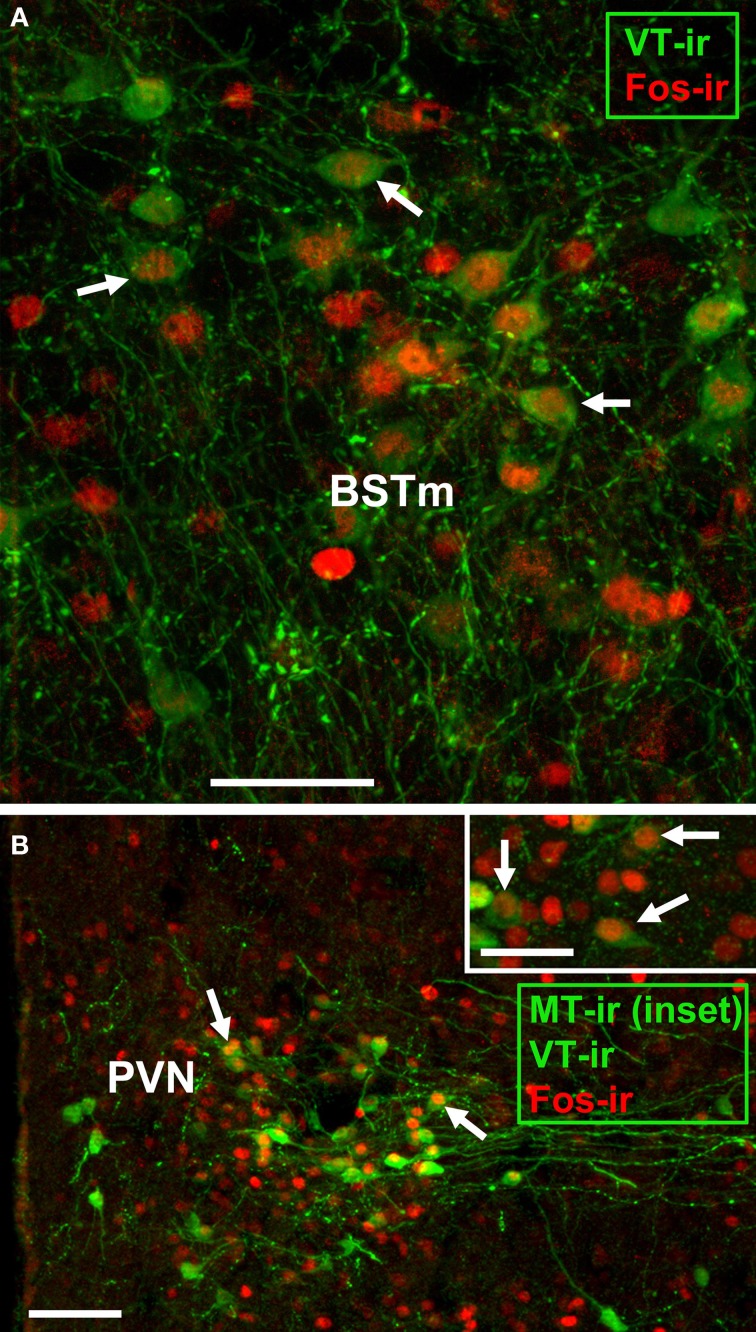
**Representative immunocytochemical colocalization of Fos with VT and MT. (A,B)** Colocalization of Fos (red; Alexa Fluor 594) and VT (green; Alexa Fluor 680 pseudocolored for visibility) in the BSTm of a male **(A)** and PVN of a female **(B)**. The inset in panel B shows colocalization of Fos (red; Alexa Fluor 594) with MT (green; Alexa Fluor 680 pseudocolored for visibility) in the PVN of a male. Scale bars = 50 μm in **(A,B)** inset; 100 μm for **(B)**. POM, medial preoptic nucleus.

In order to test the hypothesis that nonapeptide neurons respond to social stimuli in a sex- and phenotype-specific manner, we quantified expression of the immediate early gene protein Fos in nonapeptide neurons of male and female finches following interactions with familiar or novel birds. Representative photomicrographs of immunolabeling are shown in Figure 1. Because the VP-OT nonapeptides are important modulators of grouping and novel-familiar social preferences (Goodson et al., 2009d, 2012a), we hypothesized that VT and MT cell populations function differently in response to interactions with novel and familiar individuals.

Specifically, after PCA was run for all 80 birds, subjects were rank ordered by PC1, and every other bird was selected for the Fos study, with alternating assignments to treatments (novel or familiar condition). This yielded 20 birds of each sex (*n* = 10 per condition) that covered the full range of PC1 scores. For testing, all subjects were transferred to a novel cage that contained either four familiar, same-sex cagemates or four novel, same-sex individuals. Subjects were sacrificed 90 min later.

### Histology and immunocytochemsitry

Subjects were sacrificed by isoflurane overdose and perfused with 0.1 M phosphate buffered saline (PBS) followed by 4% paraformaldehyde. Brains were removed, postfixed overnight, and cryoprotected in 30% sucrose in PBS for 48 h prior to sectioning on a cryostat. Tissue was sectioned into three 40 μm series. One series was immunofluorescently labeled for VT and Fos, while a second series was labeled for MT and Fos. Tissue was rinsed 5x for 10 min in 0.1M PBS (pH 7.4), incubated for 1 h in block (PBS + 5% normal donkey serum + 0.3% Triton-X-100), and then incubated for approximately 40 h at 4°C in primary antibodies diluted in PBS containing 2.5% normal donkey serum + 0.3% Triton-X-100. Primary antibodies used for the first series were guinea pig anti-VP (1:1000; Bachem, Torrance, CA) and rabbit anti-Fos (1:1000; Santa Cruz Biotechnology, Santa Cruz, CA); primary antibodies for the second series were guinea pig anti-OT (1:1000; Bachem) and rabbit anti-Fos (1:1000; Santa Cruz Biotechnology). Note that the VP and OT antibodies generated in guinea pigs were from older Bachem lots, and we find that newer lots of these antibodies are not specific (i.e., the newer antibodies label both VT and MT). Specificity of the antibodies used here has been established (Goodson et al., 2003, 2004). The primary incubation was followed by two 30 min rinses in PBS. Tissue was incubated for 1 h in a biotinylated donkey anti-guinea pig secondary (6:1000; Jackson ImmunoResearch, West Grove, PA), rinsed twice for 15 min in PBS, and incubated for 2 h at room temperature in streptavidin conjugated to Alexa Fluor 680 (6:1000) and donkey anti-rabbit secondary conjugated to Alexa Fluor 594 (5:1000). All secondaries were diluted in PBS containing 2.5% normal donkey serum + 0.3% Triton-X-100. Alexa Fluor conjugates were obtained from Invitrogen (Carlsbad, CA). Following two 30 min rinses in PBS sections were mounted on subbed slides coverslipped with ProLong Gold antifade reagent containing DAPI nuclear stain (Invitrogen).

### Quantification and analysis

Images were acquired at 10x using a Zeiss AxioImager microscope outfitted with an AxioCam HRm, *z*-drive, and an Apotome optical dissector (Carl Zeiss Inc., Göttingen, Germany). Cell counts were conducted from flattened *z*-stacks by an observer blind to condition using Photoshop CS3 (Adobe Systems, San Jose, CA) and Image J (National Institutes of Health, Bethesda, MD), as previously described (Goodson and Wang, 2006; Goodson et al., 2009a). VT-Fos colocalization was quantified in the BSTm and PVN, at two levels at or near the level of the anterior commissure (AC), and MT-Fos colocalization was quantified in the PVN at the level of the AC and just rostral to the AC.

In our first set of analyses, which were focused on phasic Fos responses, data are expressed as the percent of VT and MT colocalizing Fos (henceforth termed VT-Fos or MT-Fos colocalization). These data were analyzed using Sex × Context ANCOVAs with one of the three behavioral PCs in each model as a covariate. This approach was taken because inclusion of Sex, Context and all three PCs in a single analysis would produce models that are over-fitted and not reliable (Forstmeier and Schielzeth, 2011). We replicated this set of analyses using the raw number of double-labeled neurons (henceforth termed VT-Fos or MT-Fos double-labeled neurons) as the dependent variable, providing a complementary view of phasic activity that is effectively weighted based on the numbers of peptide-expressing neurons.

Finally, in order to determine (1) whether peptide cell numbers (a constitutive factor) and Fos responses (a phasic factor) collectively relate to personality, and (2) whether personality relates to patterns of neuromodulation arising from multiple cell groups that may influence overlapping sets of target brain areas, we also conducted a neural PCA. This neural PCA included six variables—peptide cell numbers for each of the three neuronal populations, and the numbers of peptide neurons double-labeled for Fos in each of the three populations. We then conducted a second set of Sex × Context ANCOVAs with one of the three behavioral PCs in each model as a covariate, and one of the three neural PCs as a dependent variable. It should be noted that these analyses are intended to explore the possible interactions of personality and nonapeptide systems, and because we do not have sufficient power to conduct corrections for multiple comparisons, some positive findings might represent type 1 errors. However, far more significant effects are obtained than would be predicted by chance.

## Results

### Principal components of behavior

Behavioral phenotypes were determined for 40 male and 40 female zebra finches using assays of social preferences (group size and novel-familiar choice tests), anxiety-like behavior (novelty suppression of feeding and exploration), and observations of behavior in a colony environment, which allow for the quantification of nonsexual affiliative behaviors, aggressive behaviors, sexual behaviors, maintenance behaviors, nesting, and pair bonding. Data were initially analyzed separately for males and females using PCA (see Methods and Supplemental Tables 1, 2). However, the PC structures for males and females were very similar, and thus, in order to allow for direct comparisons of males and females in the analyses, the sexes here are combined in one PC matrix (Table 1). This required the exclusion of male-specific courtship behaviors, but this did not alter the basic PC structure. The combined analysis yields a significant model (*p* < 0.0001) containing three PCs of behavior that can be generally characterized as: Social competence/dominance (PC1; a component that may reflect individual differences in the mechanisms of social cognition), which strongly loads variables such as dominance behaviors, latency to pair bond, and preferences for familiar social partners; Gregariousness (PC2), which strongly loads only measures of group-size preference; and Anxiety (PC3), which primarily loads measures of novelty-suppressed feeding and exploration. These three PCs explain 20.3, 13.7, and 13% of the variance, respectively, and have eigenvalues of 3.040, 2.061, and 1.948. The analysis also yields four additional PCs that collectively explain 29.7% of the variance. However, we here focus only on the first three PCs because the sex-specific PCAs demonstrate strong similarities only for these more robust, first three PCs. Note that because colony interactions involved many individuals, most of the remaining variance (33.3%) is likely not intrinsic to the focal subjects. To facilitate an intuitive interpretation of our data, PC2 loadings and scores were multiplied by -1 such that higher scores are indicative of a higher level of gregariousness. Aggression data are entered separately for the first session of colony testing, when aggression is primarily focused on competition for mates, and sessions 2–6, when aggression is primarily observed in the context of nest-cup defense. Previous studies demonstrate that behavior is modulated very differently across these contexts (Kabelik et al., 2009; Goodson et al., 2012b; also see Goodson et al., 2009b).

Previous characterizations of avian phenotypes have shown that aggression and boldness are positively correlated (Drent et al., 2003; Koolhaas et al., 2007; Schurch et al., 2010). Although this is sometimes presented as a general phenomenon, the PC structure shown in Table 1 does not support that view. However, in order to directly test the relationship between anxiety-like behaviors and aggression, we ran a series of regressions between displacements exhibited in the colony test and the full range of anxiety measures. None of these analyses are significant (all *P* > 0.10).

### Fos responses of VT-MT neurons vary according to sex, social context, and personality

#### BSTm VT neurons

**Figure 2:**
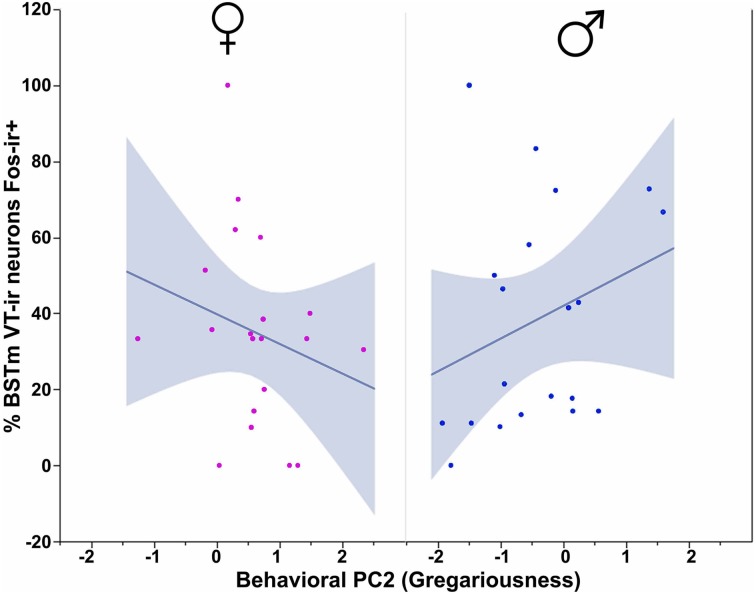
**VT-Fos colocalization in the BSTm as a function of an interaction between Sex and Behavioral PC2 (Gregariousness).** Gregariousness tends to correlate positively with VT-Fos colocalization (percent of VT neurons co-expressing Fos) in males but not females. Sex × Behavioral PC2 interaction, *F*_(1, 32)_ = 6.597, *p* = 0.02. The 95% confidence interval is indicated by shading.

**Figure 3:**
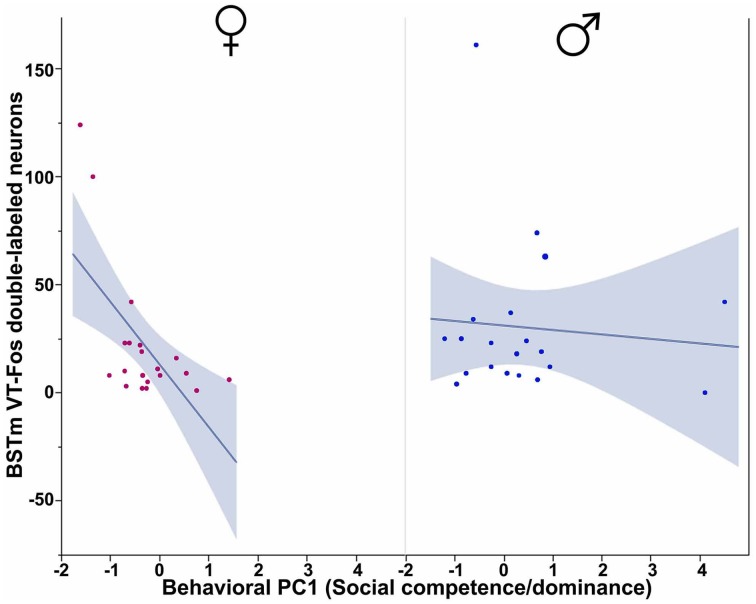
**Numbers of VT-Fos double-labeled neurons in the BSTm as a function of an interaction between Sex and Behavioral PC1 (Social competence/dominance).** Social competence/dominance tends to correlate negatively with BSTm VT-Fos double-labeled neurons in females but not males. Sex × Behavioral PC1 *F*_(1, 32)_ = 5.40, *p* = 0.03. The 95% confidence interval is indicated by shading.

In our first set of analyses, focused on the contributions of the three behavioral PCs to VT-Fos colocalization in the BSTm (i.e., percent of VT neurons colocalizing Fos), we observe a weak trend for a main effect of Behavioral PC1 (Social competence/dominance) [*F*_(1, 32)_ = 3.481, *p* = 0.07] and a significant interaction of Sex and Behavioral PC2 (Gregariousness) [*F*_(1, 32)_ = 6.597, *p* = 0.02; Figure 2]. Additional effects of Behavioral PC1 are also observed in the second set of analyses, in which the dependent variable of VT-Fos double-labeled neurons was used: main effect of Behavioral PC1 [*F*_(1, 32)_ = 7.68, *p* < 0.01], and Sex × Behavioral PC1 [*F*_(1, 32)_ = 5.40, *p* = 0.03; Figure 3].

#### PVN VT neurons

**Figure 4:**
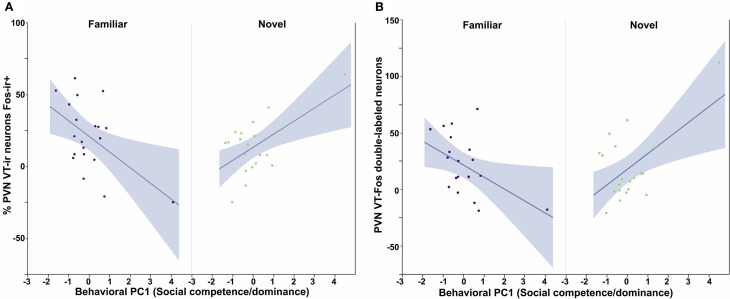
**Measures of VT-Fos co-expression in the PVN as a function of Sex and interactions between Sex and Behavioral PC1 (Social competence/dominance). (A)** Social competence/dominance tends to correlate positively with PVN VT-Fos colocalization in subjects exposed to novel same-sex conspecifics, but negatively in subjects exposed to familiar same-sex conspecifics. Context × Behavioral PC1 *F*_(1, 32)_ = 8.48, *p* < 0.01. **(B)** Similar results are obtained for the numbers of PVN VT-Fos double-labeled neurons. Context × Behavioral PC1 *F*_(1, 32)_ = 14.71, *p* < 0.001. The 95% confidence interval is indicated by shading.

**Figure 5:**
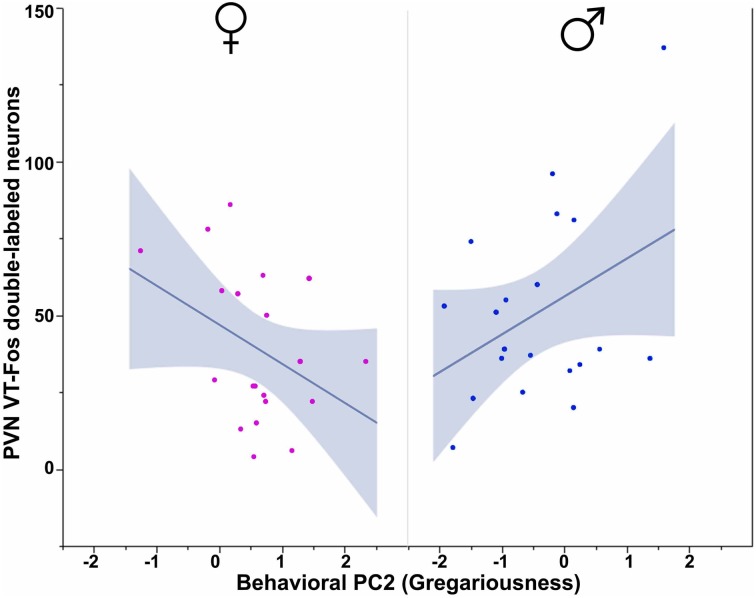
**Numbers of VT-Fos double-labeled neurons in the PVN as a function of an interaction between Sex and Behavioral PC2 (Gregariousness).** Gregariousness tends to correlate positively with PVN VT neurons expressing Fos in males, but negatively in females. Sex × Behavioral PC2 *F*_(1, 32)_ = 5.28, *p* = 0.03. The 95% confidence interval is indicated by shading.

**Figure 6:**
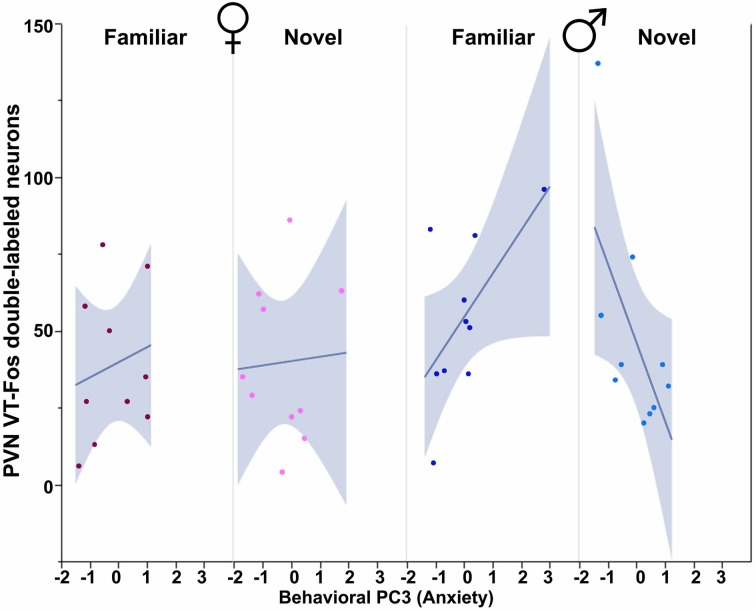
**Numbers of VT-Fos double-labeled neurons in the PVN as a function of an interaction between Behavioral PC3 (Anxiety), Sex and Context.** Anxiety tends to correlate positively with the number of PVN VT neurons expressing Fos in females and in males exposed to familiar same-sex conspecifics, but negatively in males exposed to novel conspecifics. Sex × Context × Behavioral PC3 *F*_(1, 32)_ = 4.12, *p* = 0.05. The 95% confidence interval is indicated by shading.

All three behavioral PCs are significant predictors of VT-Fos colocalization and/or double-labeling in the PVN, as are the factors of Sex and Context. In a Sex × Context × Behavioral PC1 (Social competence/dominance) analysis for VT-Fos colocalization, we observe a significant main effect of Sex [*F*_(1, 32)_ = 5.04, *p* = 0.03; females, 38.4 ± 3.5%; males, 48.4 ± 3.7%] and a significant Context × Behavioral PC1 interaction [*F*_(1, 32)_ = 8.48, *p* < 0.01; Figure 4A]. In the parallel analysis of VT-Fos double-labeled neuron numbers, we observe strong trends for the effects of Sex [*F*_(1, 32)_ = 3.72, *p* = 0.06; males > females] and Sex × Context [*F*_(1, 32)_ = 3.86, *p* = 0.06], in addition to a strong Context × Behavioral PC1 interaction [*F*_(1, 32)_ = 14.71, *p* < 0.001; Figure 4B]. Although Behavioral PC2 (Gregariousness) makes no significant contributions to the model for VT-Fos colocalization in the PVN, we nonetheless observe a significant Sex × Behavioral PC2 interaction for VT-Fos double-labeling [*F*_(1, 32)_ = 5.28, *p* = 0.03; Figure 5]. Similarly, whereas Behavioral PC3 (Anxiety) makes no significant contributions to the model for VT-Fos colocalization in the PVN, analyses of VT-Fos double-labeling yield a significant Context × Behavioral PC3 interaction [*F*_(1, 32)_ = 5.97, *p* = 0.03] and a significant Sex × Context × Behavioral PC3 interaction [*F*_(1, 32)_ = 4.12, *p* = 0.05; Figure 6].

#### PVN MT neurons

**Figure 7:**
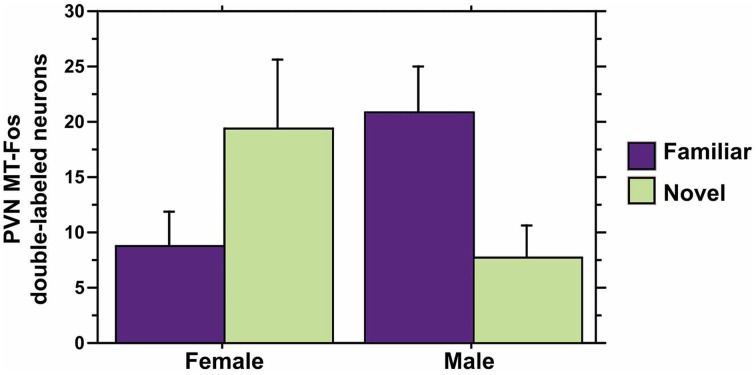
**MT-Fos double-labeled neurons in the PVN as a function of an interaction between Sex and Context.** A strong Sex × Context interaction is observed in an ANCOVA model for PVN MT-Fos double-labeled neurons with Behavioral PC1 (Social competence/dominance) as a covariate. *F*_(1, 32)_ = 8.04, *p* < 0.01. A nearly identical result is obtained with the covariate of Behavioral PC3 (Anxiety); see Results. Data are shown as means + SEM.

**Figure 8:**
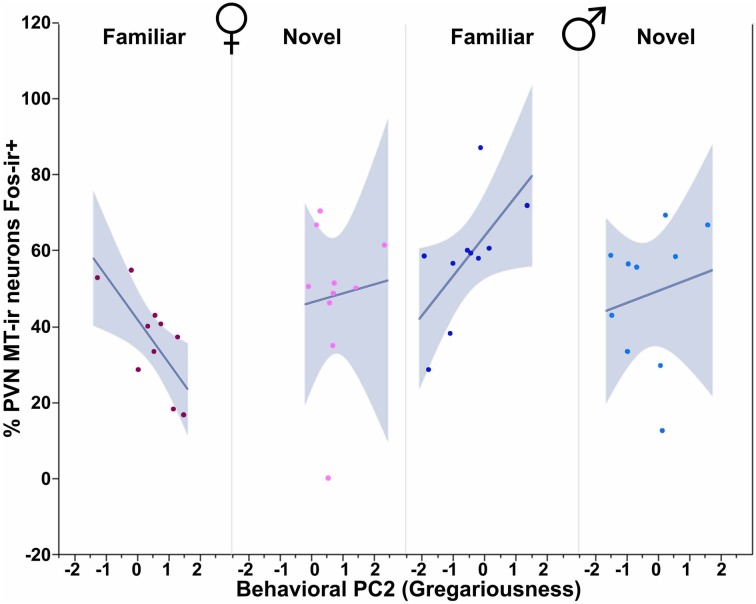
**MT-Fos colocalization in the PVN as a function of an interaction between Sex, Context and Behavioral PC2 (Gregariousness).** Gregariousness tends to correlate positively in subjects exposed to novel same-sex conspecifics, but negatively in females exposed to familiar conspecifics. Sex × Context × Behavioral PC2 *F*_(1, 32)_ = 3.88, *p* < 0.06. The 95% confidence interval is indicated by shading.

We observe significant contributions of Sex and Context to MT-Fos colocalization in most of the individual models, and some striking Sex × Context interactions, but find only weak evidence for significant PC effects. Thus, in the ANCOVA for MT-Fos colocalization that includes Behavioral PC1 (Social competence/dominance) as a covariate, we find a significant effect of Sex [*F*_(1, 32)_ = 6.29, *p* = 0.02] and a significant Sex × Context interaction [*F*_(1, 32)_ = 5.56, *p* = 0.02], but only a very weak trend for a Sex × Context × Behavioral PC1 interaction [*F*_(1, 32)_ = 3.32, *p* = 0.08]. The parallel analysis of MT-Fos double-labeled neuron numbers also yields a strong Sex × Context interaction [*F*_(1, 32)_ = 8.04, *p* < 0.01; Figure 7]. A somewhat stronger trend is observed for the contributions of Behavioral PC2 (Gregariousness) to the model for MT-Fos colocalization, where we observe a near-significant Sex × Context × Behavioral PC2 interaction [*F*_(1, 32)_ = 3.88, *p* < 0.06; Figure 8]. The ANCOVA for MT-Fos colocalization that includes Behavioral PC3 (Anxiety) as a covariate yields only a significant main effect of Sex [*F*_(1, 32)_ = 4.13, *p* = 0.05], whereas the parallel analysis of MT-Fos double-labeled neuron numbers yields a significant Sex × Context interaction [*F*_(1, 32)_ = 5.63, *p* < 0.02] that is virtually identical to the one shown in Figure 7, which includes Behavioral PC1 as the covariate.

### Neural principal components are extensively related to sex, social context and personality

Although each population of VT/VP and MT/OT neurons likely produces distinct patterns of behavioral effects, those effects must occur in the context of co-modulation by other peptide cell groups, given that the cell groups exhibit partially overlapping axonal projections and likely influence many of the same target areas through paracrine modulation (Goodson and Kabelik, 2009). The VP-OT receptors are also promiscuous (e.g., Leung et al., 2009), although the degree of this varies across species. Therefore, we conducted a PCA that includes both VT-MT cell numbers and the numbers of those neurons that were double-labeled for Fos. This yields a highly significant model (*p* < 0.0001) and three PCs that collectively explain 94.7% of the variance (Table 2). The eigenvalues for these PCs are 3.039, 1.570, and 1.072. Neural PC1 loads all variables in a positive manner. In contrast, Neural PC2 loads BSTm VT variables positively, PVN MT variables negatively, and shows very weak loadings for PVN VT variables. Only PVN VT variables load strongly on Neural PC3.

**Table 2:** **Principal components matrix of zebra finch VT-MT cell numbers and Fos co-expression^1^**.

	**PC1**	**PC2**	**PC3**
BSTm VT neurons	**0.647**	**0.698**	−0.232
BSTm VT double-labeled neurons	**0.636**	**0.731**	−0.165
PVN VT neurons	**0.778**	−0.238	0.481
PVN VT double-labeled neurons	**0.676**	0.024	**0.682**
PVN MT neurons	**0.727**	−**0.531**	−0.404
PVN MT double-labeled neurons	**0.791**	−0.457	−0.362

1 Loadings of ≥0.500 or ≤−0.500 are bolded.

**Figure 9:**
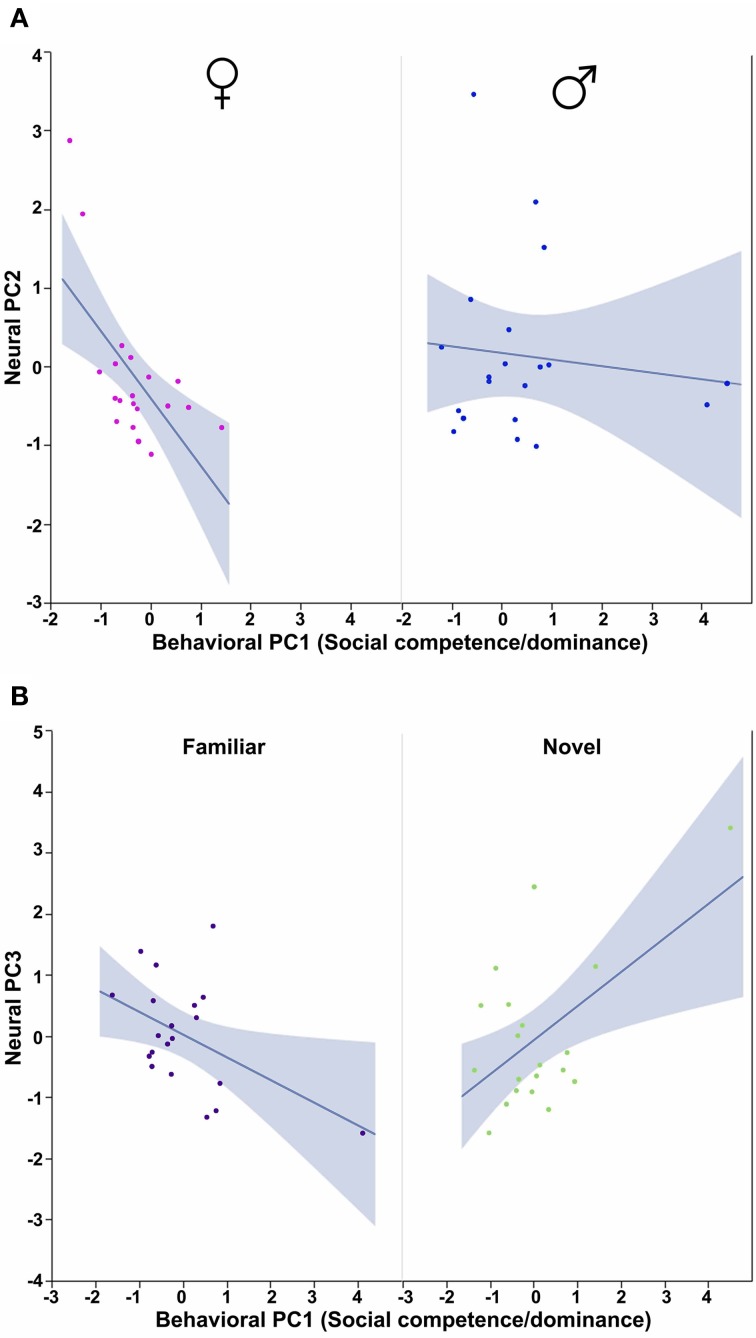
**Behavioral PC1 (Social competence/dominance) interacts with Sex to predict Neural PC2, and with Context to predict Neural PC3. (A)** The correlation between Behavioral PC1 (Social competence/dominance) and Neural PC2 is strongly negative in females but not males. Sex × Behavioral PC1 *F*_(1, 32)_ = 4.71, *p* = 0.04. **(B)** Social competence/dominance tends to correlate positively with Neural PC3 in subjects exposed to novel same-sex conspecifics, but negatively in subjects exposed to familiar conspecifics. Context × Behavioral PC1 *F*_(1, 32)_ = 13.50, *p* < 0.001. The 95% confidence interval is indicated by shading.

**Figure 10:**
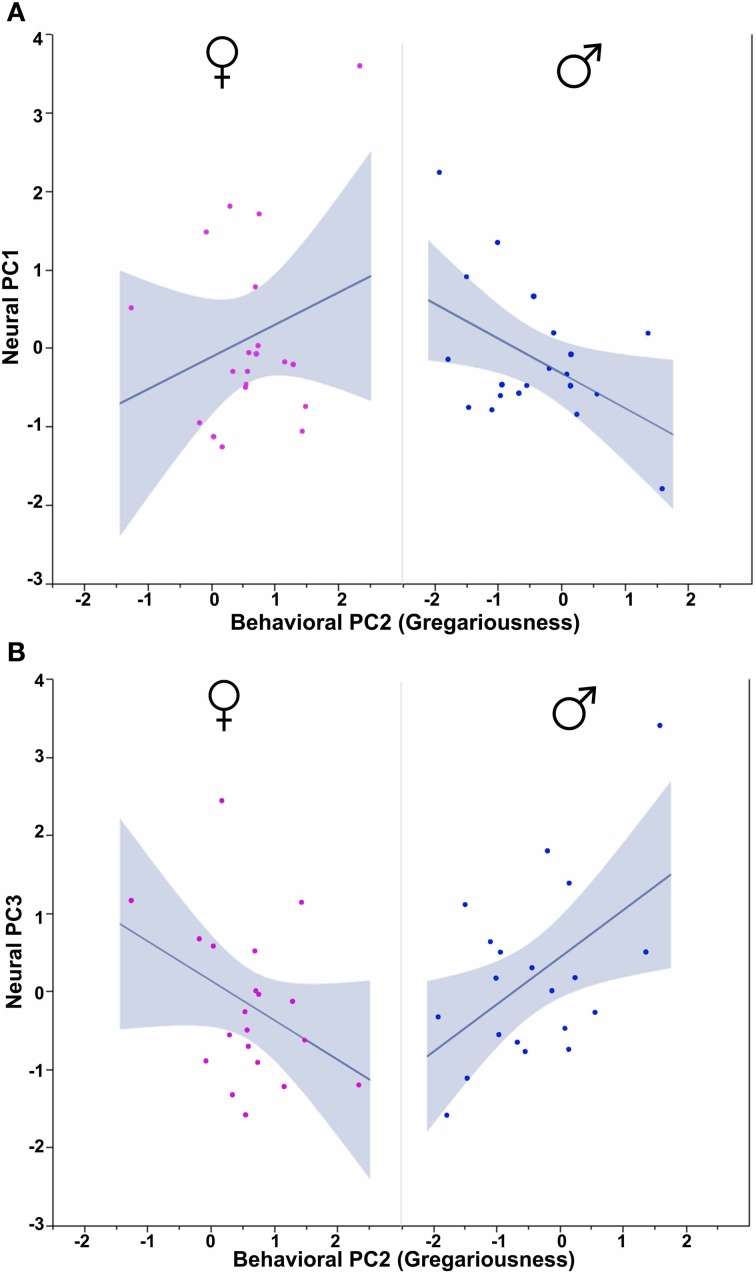
**Behavioral PC2 (Gregariousness) interacts with Sex to predict Neural PC1 and Neural PC3. (A)** PC2 (Gregariousness) tends to correlate positively with Neural PC1 in females, but negatively in males. Sex × Behavioral PC2 *F*_(1, 32)_ = 4.09, *p* = 0.05. **(B)** An opposite pattern is observed for Neural PC3, such that Gregariousness tends to correlate positively with Neural PC3 in males, but negatively in females. Sex × Behavioral PC2 *F*_(1, 32)_ = 7.28, *p* = 0.01. The 95% confidence interval is indicated by shading.

**Figure 11:**
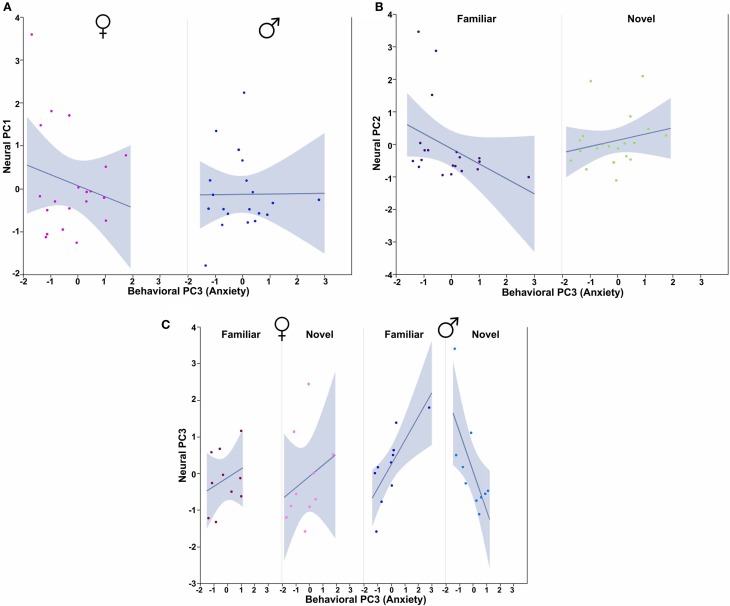
**Behavioral PC3 (Anxiety) interacts with Sex and Context to predict multiple Neural PCs. (A)** PC3 (Anxiety) tends to correlate negatively with Neural PC1 in females, but not males. Sex × Behavioral PC3 *F*_(1, 32)_ = 4.01, *p* = 0.05. **(B)** Anxiety tends to correlate positively with Neural PC2 in subjects exposed to novel conspecifics, but negatively in subjects exposed to familiar conspecifics. Context × Behavioral PC3 interaction *F*_(1, 32)_ = 3.49, *p* = 0.07. **(C)** Anxiety tends to correlate positively with Neural PC3 in females and males exposed to familiar conspecifics, but negatively in males exposed to novel conspecifics. Sex × Context × Behavioral PC3 *F*_(1, 32)_ = 7.76, *p* < 0.01. The 95% confidence interval is indicated by shading.

In order to determine how the three behavioral PCs, Sex and Context relate to these neural PCs, we conducted Sex × Context ANCOVAs, each of which included a single behavioral PC as a covariate and one of the three neural PCs as a dependent variable. These analyses reveal strong contributions of Sex, Context and all three behavioral PCs. Notably, each behavioral PC tends to relate to a distinct subset of neural PCs. As described more fully in the results below, Behavioral PC1 (Social competence/dominance) relates primarily to Neural PC2 and Neural PC3 (Figure 9), whereas Behavioral PC2 (Gregariousness) relates primarily to Neural PC1 and Neural PC3 (Figure 10). As with Behavioral PC2, Behavioral PC3 (Anxiety) exerts significant effects in the models for Neural PC1 and Neural PC3, but it also exerts a marginal effect on Neural PC2 (Figure 11).

The ANCOVA model for Neural PC2 with Behavioral PC1 (Social competence/dominance) as a covariate reveals a significant main effect of Behavioral PC1 [*F*_(1, 32)_ = 7.04, *p* = 0.01] and a significant Sex × Behavioral PC1 interaction [*F*_(1, 32)_ = 4.71, *p* = 0.04; Figure 9A]. In addition, the comparable model for Neural PC3 yields a very strong Context × Behavioral PC1 interaction [*F*_(1, 32)_ = 13.50, *p* < 0.001; Figure 9B].

Using Behavioral PC2 (Gregariousness) as a covariate in a model for Neural PC1, we observe a significant Sex × Behavioral PC2 interaction [*F*_(1, 32)_ = 4.09, *p* = 0.05; Figure 10A], and in the comparable model for Neural PC3, we also find a significant Sex × Behavioral PC2 interaction [*F*_(1, 32)_ = 7.28, *p* = 0.01; Figure 10B].

Finally, using Behavioral PC3 (Anxiety) as a covariate in a model for Neural PC1, we observe a significant Sex × Behavioral PC3 interaction [*F*_(1, 32)_ = 4.01, *p* = 0.05; Figure 11A]. The comparable model for Neural PC2 shows a trend for a Context × Behavioral PC3 interaction [*F*_(1, 32)_ = 3.49, *p* = 0.07; Figure 10B], and the comparable model for Neural PC3 yields significant interaction effects for both Context × Behavioral PC3 [*F*_(1, 32)_ = 6.63, *p* = 0.01] and Sex × Context × Behavioral PC3 [*F*_(1, 32)_ = 7.76, *p* < 0.01; Figure 11C].

## Discussion

The goals of the present experiment were two-fold. First, we wished to elucidate the structure of complex behavioral phenotypes (personalities) in zebra finches, and second, we wanted to use that structure as a tool to probe phenotype-specific functions of nonapeptide cell groups that are important for social behavior. We find that Fos responses of VT-MT cells are extensively predicted by sex, social context, and multiple dimensions of personality. Furthermore, we observe many relationships between sex, context and personality that reflect complex interactions across the cell groups of the PVN and BSTm.

### Personality structure in zebra finches

A complication of comparing human and nonhuman animal personalities is that the construction of human personalities is largely based on verbal and written questionnaires and self-reports, whereas assays in nonhuman animals rely upon the quantification of overt behaviors. Nonetheless, some similarities are observed across species. In humans, 5 trait dimensions of personality are generally recognized that include Neuroticism (or emotional stability), Agreeableness (friendliness and sociability), Extraversion (including dominance), Openness (including openness to experience), and Conscientiousness (or constraint) (John, 1990), and despite some controversy relating to anthropomorphism (Gosling and Vazire, 2002; Bell, 2007; Reale et al., 2007), somewhat similar trait dimensions have been observed in nonhuman animals. For example, factor analysis of behavioral data in wild male crested macaques (*Macaca nigra*) reveal a 4-factor structure with components that can be characterized as Anxiety, Sociability, Connectedness, and Aggressiveness (Neumann et al., 2013). Other studies in cats, dogs and a variety of primates reveal personality traits that are similar to the human factors of Neuroticism, Agreeableness, Extraversion, and Openness, often with two additional dimensions of Dominance and Activity (Gosling and John, 1999).

To date, similarly complex personality profiles have not been generated for other species that are more tractable for neurobiological experiments, although numerous taxa such as rodents, birds and fish have been studied in relation to exploratory behavior and responses to novelty, which are often presented as measures of “bold/shy” behavior. Given that bold/shy behavior represents only one trait dimension, it is of questionable utility to present it as “personality,” given that personality is classically defined in a much more complex way within the field of psychology. However, in some studies this trait dimension has been additionally linked to various aspects of social behavior (Pike et al., 2008; Croft et al., 2009; Fox et al., 2009; Scheutt and Dall, 2009; Aplin et al., 2013), stress coping (Groothuis and Carere, 2005; Koolhaas et al., 2010), task specialization (in social insects) (Grinsted et al., 2013), and extrapair paternity (Van Oers et al., 2008), which places the bold/shy continuum in a somewhat richer phenotypic context.

In the present paper we took an approach for defining dimensions of personality in zebra finches that is similar to the methods employed for primates. We quantified a total of 23 social and nonsocial behaviors and analyzed the resulting data using PCA. Three main components resulted from the PCA, which we characterize as Social competence/dominance, Gregariousness, and Anxiety. These dimensions of personality in zebra finches are somewhat similar to personality factors in primates, although the structure of personality is clearly species-specific, as should be expected. Of particular interest in the context of species-specificity are (1) the structure of PC1, which links pair bonding (obviously restricted to monogamous species) to dominance behavior, and (2) the structure of PC2, a component that is essentially defined by the preference for larger or smaller groups (a trait that is meaningful only in species that form groups). Importantly, if PC2 simply represented social contact behavior, then time with the large group and time with the small group should both load positively, but this was not the case; rather, these variables load very highly, but in opposite directions.

Also of interest in relation to species-specificity is the clear lack of a correlation between PC3 (Anxiety) and aggressive behavior. Other investigators have noted an association between these variables (Drent et al., 2003; Koolhaas et al., 2007; Schurch et al., 2010) and have therefore described animals as proactive and reactive (Aplin et al., 2013), rather than simply bold and shy. However, this association appears to vary according to species, suggesting caution when generalizing.

### Functional profile of BSTm VT cells

Although a great deal is known about the behavioral functions of the OT-VP peptides, relatively few investigations have linked these functions to specific cell groups. Most of the behaviorally relevant data for the BSTm VT/VP cells come from immediate early gene experiments, which demonstrate that these neurons exhibit an increase in transcriptional activity (i.e., Fos immunoreactivity) selectively in response to positive social stimuli, as shown in several finch species (Goodson and Wang, 2006), and that similarly, activation of these neurons is associated with appetitive sexual behavior but not agonistic behavior in chickens (Xie et al., 2011), and copulation but not aggressive interactions in mice (Ho et al., 2010). The percent of BSTm VT cells expressing Fos also correlates with the intensity of male sexual behavior in brown anoles (*Anolis sagrei*), but not with the intensity of male-male aggression (Kabelik et al., 2013). Similarly, overnight cohabitation with a female increases VP mRNA in the BSTm of male prairie voles (Wang et al., 1994). Finally, RNA interference experiments in zebra finches provide direct evidence that BSTm VT neurons promote gregariousness and suppress aggression in a male-specific manner; promote male courtship singing; and reduce anxiety-like behavior in both males and females (Kelly et al., 2011; Kelly and Goodson, 2013b). Somewhat comparable results are obtained in the modestly gregarious Angolan blue waxbill (*Uraeginthus angolensis*), in which VT knockdown reduces social contact more strongly in males than in females (Kelly and Goodson, 2013a).

Consistent with the male-specific antisense effects on gregariousness in zebra finches (Kelly et al., 2011; Kelly and Goodson, 2013b), we here find that VT-Fos colocalization correlates positively with Gregariousness (PC2) only in males (as shown in Figure 2). However, we were surprised to find no relationship between VT-Fos labeling in the BSTm and PC3 (Anxiety), given that anxiety is increased in both male and female zebra finches following VT knockdown in the BSTm (Kelly et al., 2011; Kelly and Goodson, 2013b). It is possible that PC3 interacts with other personality dimensions to predict VT-Fos co-expression, and/or that phenotypic variation in anxiety arises through an interaction of VT circuitry arising from the BSTm with nonapeptide circuits arising in the PVN, which may impinge upon some of the same postsynaptic targets. As addressed in the final section below, this may well be the case.

### Functional profile of PVN VT cells

To our knowledge, social behavior has not been quantified following direct manipulations of the VT/VP cell group in the PVN, or homologous cells in anamniotes, which lie in the POA (Goodson and Bass, 2001) (although we are preparing such a dataset in zebra finches). However, across all vertebrates, these neurons innervate the anterior pituitary where VT/VP synergizes with corticotropin releasing hormone to stimulate the release of adrenocorticotropic hormone (Rivier and Vale, 1983; Baker et al., 1996; Cornett et al., 2013). Thus, PVN VT/VP neurons and their anamniote homologs play important roles in glucocorticoid tone and physiological responses to stress. Consistent with this role in stress response, VT/VP expression in the PVN increases following a variety of social and nonsocial stressors (Veenema and Neumann, 2009; Murakami et al., 2011; Zhang et al., 2012), and subordinate male mice exhibit significantly greater Fos induction in PVN VP neurons following aggressive interactions than do dominant individuals (Ho et al., 2010). Similarly, VT-Fos colocalization is negatively correlated with aggression in territorial song sparrows (*Melospiza melodia*) (Goodson and Evans, 2004). In fish, parvocellular VT neurons are likewise associated with subordination and social avoidance (Greenwood et al., 2008). However, a different pattern of results is obtained in male anoles, in which both sexual behavior and aggression correlate *positively* with VT-Fos colocalization in the PVN (Kabelik et al., 2013). This difference may reflect species-specific relationships between VT/VP and behavior, or alternatively may reflect the fact that the social tests in anoles induced a stress response.

The present results demonstrate that the Fos activity of PVN VT neurons reflects anxiety phenotype, as hypothesized based on their roles in stress response, although this relationship is complex, as PC3 (Anxiety) interacts with both Sex and Context to predict VT-Fos double-labeling. We also observe an interaction of Sex and PC2 (Gregariousness) for VT-Fos co-expression that is very similar to that obtained for the BSTm; i.e., that co-expression is positively correlated with gregariousness in males but not females. Interestingly, males of sparrow species that flock in winter exhibit significantly more VT-ir neurons in the PVN than do species that do not flock, and this difference is not observed during the spring, when all of the species are territorial (Goodson et al., 2012c). Hence, converging lines of evidence from the finch and sparrow families suggest that PVN VT neurons relate in a positive manner to male gregariousness and flocking.

### Functional profile of PVN MT cells

Only one study that we are aware of has directly manipulated OT neurons in the PVN. While this study did not examine the effects of OT neurons on affiliation behaviors, it demonstrated that acute reduction of OT synthesis via antisense administration increases maternal aggression in postpartum rats (Giovenardi et al., 1998). This finding is consistent with numerous studies showing that OT has profound effects on maternal behaviors (including maternal aggression) and affiliation (Bosch et al., 2005; Carter et al., 2008; Neumann, 2009; Goodson and Thompson, 2010). Release of OT in the region of the PVN (presumably of local origin) also promotes parturition and maternal behavior (Da Costa et al., 1996). In addition, endogenous OT receptor (OTR) activation in multiple brain areas (e.g., PVN and central amygdala) is associated with anxiolysis and fear reduction (McCarthy et al., 1996; Windle et al., 1997; Bale et al., 2001; Knobloch et al., 2012); and PVN OT neurons project to extrahypothalamic brain areas and the spinal cord where OT promotes copulatory behavior and penile erection in male mammals (Argiolas and Melis, 2004). OT and homologous peptides are also associated with affiliation in nonreproductive contexts. For instance, social interaction in mice positively correlates with OT mRNA expression in the PVN (Murakami et al., 2011), and isotocin promotes social approach toward novel, same-sex conspecifics in goldfish (Thompson and Walton, 2004).

In zebra finches, endogenous OTR activation and exogenous MT infusions promote gregariousness and a preference for familiar same-sex individuals (Goodson et al., 2009d). Consistent with these findings, we here find that PVN MT-Fos colocalization and MT-Fos double-labeling is extensively predicted by Sex and Context (novel vs. familiar), and that double-labeling is predicted in a near-significant manner by an interaction of Sex, Context and Behavioral PC2 (Gregariousness). In addition, PVN MT cell numbers and MT-Fos double-labeling load strongly onto neural PCs that are extensively predicted by all three behavioral PCs, Sex and Context, as addressed in the next section. Findings in humans likewise suggest that OT modulation varies in relation to sex, context, and personality. For instance, plasma OT levels correlate positively with Extraversion scores (Andari et al., 2012), and OT nasal spray produces neural and behavioral responses in the Prisoner's Dilemma game that are strongly sexually differentiated (Rilling et al., 2013). A variety of other data further suggest that OT effects on human behavior are dependent upon situational variables (context) as well as individual personality (Bartz et al., 2011).

### Neuromodulatory patterning

As suggested by the findings discussed above, each of the VT-MT cell groups likely exerts distinct suites of behavioral effects. To some extent this may be surprising, given that peptide derived from the BSTm and PVN cell groups likely reaches many of the same brain targets, either through paracrine action or direct innervation (Goodson and Kabelik, 2009). Avian nonapeptide receptors are also highly promiscuous (Leung et al., 2009), increasing the potential for the convergence of MT and VT signaling. Nonetheless, the overall spatial pattern of modulation in the brain (the “neuromodulatory pattern”) that is produced by each cell group is likely unique, and this should yield distinct behavioral outcomes (Goodson and Kabelik, 2009), as suggested by the present results.

At the same time, however, the extensive overlap of signaling suggests that sex-, context-, and phenotype-specific variation in behavior may be extensively dependent upon the interactions between peptide circuits arising from peptide neurons of the BSTm and PVN. In order to test this idea, we conducted a neural PCA of numbers of VT and MT neurons (a constitutive feature) and the numbers of those neurons that colocalize Fos. This analysis yielded three PCs that collectively account for 94.7% of the variance. Neural PC1 loads all variables positively, suggesting that this PC may reflect consistent and collective modulation by VT-MT cell groups on target brain areas. Even so, Neural PC1 is predicted by interactions of Sex with both Behavioral PC2 (Gregariousness) and Behavioral PC3 (Anxiety). Neural PC2 has a very different structure—loading BSTm VT variables positively and PVN MT variables negatively. In contrast to Neural PC1, this neural PC is predicted by an interaction of Sex with Behavioral PC1, and in a manner that suggests a positive relationship between BSTm VT neurons (relative to PVN MT neurons) and Social competence/dominance in males, and a positive relationship between PVN MT neurons (relative to BSTm VT neurons) and Social competence/dominance in females. Neural PC2 also shows a weak relationship to Behavioral PC3 (Anxiety), but is not significantly predicted by Behavioral PC2 (Gregariousness). Finally, Neural PC3 loads PVN variables positively and shows only weak loadings for other variables. This PC is extensively predicted by a variety of variables, including interactions of Context and Behavioral PC1 (Social competence/dominance); Sex and Behavioral PC2 (Gregariousness); and a complex interaction of Sex, Context and Behavioral PC3 (Anxiety). In the end, Sex, Context, and each dimension of personality relate in a unique way to the three neural PCs, suggesting that a broad, simultaneous focus on multiple peptide cell groups may be more informative than analyses of individual populations.

## Conclusions

The VP-OT nonapeptides are major points of focus in behavioral neuroscience, and many studies have now documented variation in these systems (e.g., in function, anatomy, and gene expression) that reflects sex, individual differences, and social context. These observations suggest that personality dimensions may predict the socially induced responses of nonapeptide neurons, including sex- and context-specific responses, which we here confirm. The effects observed for some cell groups are strongly consistent with other experimental data. However, the extent of the interactions between personality dimensions, Sex and Context were unexpected, suggesting that we have much to learn about the biology of nonapeptide systems. Even more notable is the finding that these variables are each related in a unique way to a set of neural PCs, suggesting that phenotypic and contextual variation are strongly tied to variable patterns of interactions across peptide circuits arising from the BSTm and PVN.

## Author contributions

Aubrey M. Kelly conducted the experiments. Both authors designed the experiments, analyzed the data, and wrote the paper.

### Conflict of interest statement

The authors declare that the research was conducted in the absence of any commercial or financial relationships that could be construed as a potential conflict of interest.

## Supplementary material

Supplemental Table 1 **Principal components matrix of male zebra finch behavior**.

Supplemental Table 2 **Principal components matrix of female zebra finch behavior**.
