# Supplementary material for: Personality is tightly coupled to vasopressin-oxytocin neuron activity in a gregarious finch
Source: Front Behav Neurosci. 2014 Feb 25;8:55. doi: 10.3389/fnbeh.2014.00055 (PMC3933816; doi:10.3389/fnbeh.2014.00055)
Supplement: Supplemental Table 1 — Principal components matrix of male zebra finch behavior. [file DataSheet1.DOCX]

Supplemental Table 1. Principal components matrix of male zebra finch behavior

|  | **PC1** | **PC2**^1^ | **PC3**^1^ |
| --- | --- | --- | --- |
| **Behavioral Measures** |  |  |  |
| *Colony Behaviors* |  |  |  |
| displacements (s1) ^2^ | 0.058 | **0.616** | -0.248. |
| displacements (s2-6) ^2^ | **0.794** | 0.253 | -0.016. |
| displaced by others (s1) ^2^ | -0.453**-** | -0.194**-** | 0.011 |
| displaced by others (s2-6) ^2^ | **-0.676-** | 0.011 | -0.036**-** |
| threats | **0.726** | 0.016 | 0.194 |
| latency to pair bond | **-0.578-** | 0.170 | -0.337. |
| time on nest | **0.786** | -0.148**-** | 0.201 |
|  |  |  |  |
| *Choice tests* |  |  |  |
| time with novel birds | **-0.638-** | 0.389 | 0.140 |
| time with familiar birds | **0.595** | -0.064. | 0.201 |
| time with small group | -0.066. | 0.245 | **0.821** |
| time with large group | 0.234 | -0.271. | **-0.593.** |
|  |  |  |  |
| *Anxiety tests* |  |  |  |
| latency to move (feed) | 0.263 | 0.353 | **-0.550**. |
| latency to feed | 0.395 | **0.606** | -0.372. |
| latency to move (explore) | -0.015. | **0.690** | 0.122 |
| branches explored | 0.108 | **-0.651.** | -0.269- |

^1^ Note that in this matrix for males, PC2 primarily loads anxiety variables and PC3 primarily loads variables related to gregariousness. This order is reversed from the matrix with the sexes combined (Table 1).

^2^ Aggression data for the first colony session (s1) was quantified separately from aggression data in the subsequent 5 sessions (s2-6) because aggression is initially focused on competition for mates and is later focused on nest defense.

Variance Proportion: PC1 = 0.255; PC2 = 0.147; PC3 = 0.125

Supplemental Table 2. Principal components matrix of female zebra finch behavior

|  | **PC1** | **PC2** | **PC3** |
| --- | --- | --- | --- |
| **Behavioral Measures** |  |  |  |
| *Colony Behaviors* |  |  |  |
| displacements (s1)^1^ | 0.495 | -0.165**.** | -0.165. |
| displacements (s2-6)^1^ | **0.659** | -0.055. | 0.228 |
| displaced by others (s1)^1^ | -0.095**-** | 0.416 | 0.207 |
| displaced by others (s2-6)^1^ | -0.249**-** | 0.434 | **-0.553-** |
| threats | 0.491 | 0.491 | 0.138 |
| latency to pair bond | **-0.827-** | -0.177. | 0.172 |
| time on nest | **0.690** | 0.199 | 0.111 |
|  |  |  |  |
| *Choice tests* |  |  |  |
| time with novel birds | -0.060- | 0.402 | -0.342. |
| time with familiar birds | -0.483. | 0.037 | 0.496 |
| time with small group | 0.097 | **-0.502.** | **-0.568.** |
| time with large group | -0.202. | **0.626** | **0.626** |
|  |  |  |  |
| *Anxiety tests* |  |  |  |
| latency to move (feed) | -0.409. | -0.447. | -0.096. |
| latency to feed | -0.417. | **-0.522.** | 0.181 |
| latency to move (explore) | 0.337 | -0.464**.** | **0.504** |
| branches explored | -0.269. | **0.527** | -0.289- |

^1^ Aggression data for the first colony session (s1) was quantified separately from aggression data in the subsequent 5 sessions (s2-6) because aggression is initially focused on competition for mates and is later focused on nest defense.

Variance Proportion: PC1 = 0.198; PC2 = 0.152; PC3 = 0.130
